# Ultrasonography-guided closed reduction in the treatment of displaced transphyseal fracture of the distal humerus

**DOI:** 10.1186/s13018-020-02118-2

**Published:** 2020-12-01

**Authors:** Hai Zhou, Ge Zhang, Ming Li, Xiangyang Qu, Yujiang Cao, Xing Liu, Yuan Zhang

**Affiliations:** 1 Department of Orthopaedics, Children’s Hospital of Chongqing Medical University, 136 Zhongshan Er Road, Yuzhong District, Chongqing, 400014 People’s Republic of China; 2 Ministry of Education Key Laboratory of Child, Children’s Hospital of Chongqing Medical University, 136 Zhongshan Er Road, Yuzhong District, Chongqing, 400014 People’s Republic of China; 3 Development and Disorders, Children’s Hospital of Chongqing Medical University, 136 Zhongshan Er Road, Yuzhong District, Chongqing, 400014 People’s Republic of China; 4 National Clinical Research Center for Child Health and Disorders, Children’s Hospital of Chongqing Medical University, 136 Zhongshan Er Road, Yuzhong District, Chongqing, 400014 People’s Republic of China; 5 China International Science and Technology Cooperation base of Child development and Critical Disorders, Children’s Hospital of Chongqing Medical University, 136 Zhongshan Er Road, Yuzhong District, Chongqing, 400014 People’s Republic of China

**Keywords:** Ultrasonography, Closed reduction, Displaced transphyseal fracture of the distal humerus

## Abstract

**Background:**

To evaluate the clinical and radiographic outcomes of ultrasonography-guided closed reduction in the treatment of displaced transphyseal fracture of the distal humerus (TFDH).

**Methods:**

Twenty-seven patients with displaced TFDH were successfully treated by the ultrasonography-guided closed reduction during January 2012 to December 2016 and were retrospectively reviewed. After the mean follow-up of 34.88 months, the clinical and radiographic outcomes of patients were evaluated. The cubitus varus of the affected elbows was also assessed at the latest follow-up.

**Results:**

The successful rate of ultrasonography-guided closed reduction in the treatment of displaced TFDH was 84% (27/32). The twenty-seven patients with successful reduction were included for the following analysis. There were 20 males and 7 females included in the study, and the mean age at treatment was 15.39 ± 3.10 months; seventeen fractures occurred in the right side elbow and ten in the left side. At the last follow-up, there were significant decreases in the elbow flexion (3°, *P* = 0.027) and range of motion (5°, *P* = 0.003) between the injured and uninjured elbow, respectively, whereas no difference in elbow extension was detected (*P* = 0.110). Flynn’s criteria assessment showed that all the patients achieved excellent or good outcomes both in the functional and cosmetic categories. The clinical and radiographic carrying angles at the last follow-up were 11.67 ± 3.11° and 10.46 ± 3.88°, respectively. And the incidence of cubitus varus after treatment was 7.4% at the last follow-up.

**Conclusion:**

The ultrasonography-guided closed reduction in the treatment of displaced TFDH is an effective procedure; the adequate fracture reduction can be acquired with the advantages of real-time, non-radioactive, and simple utilization. With the percutaneous pining fixation, satisfactory clinical and radiographic outcomes can be achieved with a low incidence of postoperative cubitus varus.

## Introduction

The transphyseal fracture of the distal humerus (TFDH) is a rare and occurring in young children below age of 3 years old [1]. Owing to the unossification of most epiphyseal centers of elbow at this age, most of the distal humeral epiphysis cannot be visualized directly on radiography, or only the capitellum ossification center can be seen [2, 3]. Nowadays, as the deep understanding of TFDH, it is sufficient to make diagnosis according to the patient’s age and the typical radiographic manifestations that posteromedial displacement of proximal radio-ulnar complex is relative to the distal humeral metaphysis [4].

Because of the low incidence of TFDH, many previous studies just reported case reports on this injury with limited impacts on the treatment of TFDH. Recently, some studies reported retrospective studies with small number of consecutive cases [5, 6]. However, the consensus on the normalized treatment has not been reached, and it is still a need of further seeking on the most appropriate treatment strategy for the TFDH. The open reduction enabled the fracture reduced under the direct vision that can ensure the approximately anatomic reduction. However, there are some inevitable risks involved, such as wound infections, bleeding, and local scars [7]. Closed reduction can avoid most complications related to the open incision, as the closed reduction and percutaneous pining fixation has been established as the conventional procedure in treating displaced supracondylar fractures [8]. Closed reduction with percutaneous K-wire fixation has also become a widely acceptable technique in the treatment of the TFDH [9]. As the closed reduction needs to be assisted by the X-ray fluoroscopy, a great quantity of X-ray is commonly unavoidable to achieve accurate reduction and realignment, which will be resulting in considerable radiation exposure to both surgical staff and patients. Nevertheless, intraoperative elbow arthrography is necessary when treating TFDH with closed reduction which is an invasive procedure with a risk of infection [10].

The ultrasonography (US) is a non-invasive technique that is well-tolerated by children of all ages. The high-resolution transducer depicts internal musculoskeletal structures well, which are generally sufficient in infants and young children [11]. Some case reports have introduced the utilization of US in diagnosis, treatment, and assessment of the TFDH [12, 13]. To our best knowledge, there is no research on the consecutive cases about the intraoperative US-guided reduction of TFDH. In this work, we describe a procedure which uses intraoperative US to assist closed reduction of displaced TFDH and further evaluate the clinical and radiographic outcomes, retrospectively.

## Methods

### Patients selection

After institutional review board approval by Children’s Hospital of Chongqing Medical University, we screened patients with the diagnosis of the TFDH from January 2012 to December 2016 to in our single tertiary medical institution. Our inclusion criteria were (1) patients who were initially diagnosed with transphyseal fracture of the distal humerus (we reviewed all the patients’ preoperative radiographic data to identify the diagnosis); (2) patients should have undergone closed reduction guided by US as the first choice; and (3) patients whose radiographic data were followed for at least 24 months. The exclusion criteria were (1) patients who have been diagnosed with TFDH in the database, wherein the diagnosis of TFDH was excluded after a thorough radiographic screen; (2) patients with a duration from injury to treatment of more than 5 days; (3) patients who did not undergo closed reduction as the first treatment choice; (4) patients who underwent reduction without US guidance; (5) patients whose follow-up duration was less than 24 months; (6) patients with incomplete clinical and radiographic data at presentation; and (7) patients whose age at the treatment was more than 3 years old.

### Surgical technique

**Fig. 1:**
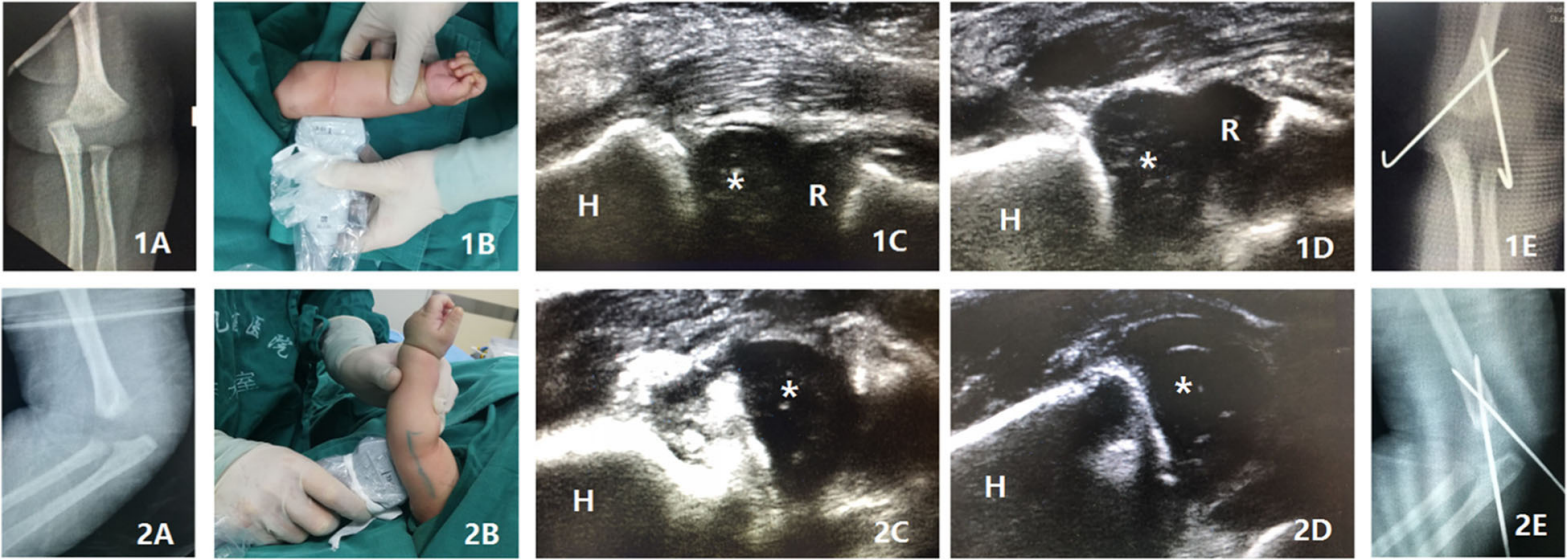
An 9-month-old boy with TFDH secondary to falling down from a bed. The typical medial displacement of the proximal forearm related to the distal humerus on the anteroposterior radiography (**1A**). The ultrasonic transducer was placed to the radial side of the elbow to assess the lateral displacement of the fracture (**1B**). The medial displacement of the humeral capitellum (*) and the radial head (R) compared with the humerus (H) (**1C**). After the closed reduction, the relationship between humeral capitellum (*) and humerus (H) has been corrected (**1D**). The anteroposterior radiography after treatment (**1E**). The typical posterior displacement of the proximal forearm related to the distal humerus on the lateral radiography (**2A**). The transducer was placed to the posterior side of the distal humerus to assess the posterior displacement of the distal humerus (**2B**). The posterior displacement of the humeral capitellum (*) compared with humerus (H) (**2C**). After the closed reduction, the posterior displacement of the humeral capitellum (*) has been corrected (**2D**). The lateral radiography after treatment (**2E**)

The procedure was performed under general anesthesia, after reassurance of the fracture displacement of the affected elbow by C-arm fluoroscopy. The high-resolution ultrasonography (SonoSite, 5-10 MHz; Inc., Bothell, WA) was used to guide closed reduction intraoperatively. The transducer was coated with a sterile endoscope cover. And the iodophor was used as an ultrasonic couplant. During reduction manipulation, ultrasound imaging of the distal humerus was performed in two standardized longitudinal sectional planes which were the radial/lateral side and the posterior side. The radial side ultrasonography was used to show the lateral displacement of distal end of the fracture; meanwhile, the posterior ultrasonography was used to show the posterior displacement of the distal end of the fracture. The reduction maneuvers were similar to those used for supracondylar fractures. Briefly, all the patients were in the supine position; we first corrected the lateral displacement by pushing the distal fracture fragment under the gentle traction with elbow in extension. The ultrasonic transducer was placed to the radial side of the elbow to assess the correction of the lateral displacement of the fracture. Next, the elbow was flexed while pushing the olecranon with the thumb to correct the posterior displacement of the distal humeral epiphysis. At the same time, the forearm was pronated or supinated to correct the rotation of the fragment. The ultrasonic transducer was placed to the posterior side of the distal humerus to evaluate the posterior displacement of the distal fracture end (Fig. 1). Once the acceptable reduction of fracture had been achieved, the elbow was maintained in the maximum flexion to stabilize the reduction and fixation by two K-wires (1.4 mm–1.6 mm in diameter) through percutaneous pinning with the crossed-pin configuration. And the fracture reduction was further confirmed through radiography, which was manifested as the corrected relationship between the distal humeral and forearm. After assurance of the reduction and stablity, the pins were then bent and cut, the arm was placed in long arm casting, and the child was awoken from anesthesia. The long-arm casting plaster was utilized to assist the immobilization of the fracture till removal of the internal fixation pins when fracture healing was documented on two views in the outpatient clinic, usually at 4–6 weeks postoperatively.

### Follow-up

Elbow plain film was taken postoperatively immediately, at 2, 4, and 6 weeks after operation, respectively. Thereafter, patients were followed at the interval of 3 months till the last follow-up.

### Clinical evaluation

The clinical outcomes of patients at the last follow-up were evaluated using Flynn’s criteria. The flexion and extension ranges as well as the carrying angles of both affected and unaffected elbows were assessed with the utilization of a goniometer [14]. The carrying angle defined the angle formed by the long axis of the upper arm and the long axis of the forearm in the frontal plane which measured with the elbow extended and the forearm and hand in full supination.

### Radiographic evaluation

The radiographic outcomes were evaluated on anterioposterior and lateral radiographs of both injured and unaffected elbows at the last follow-up among all the patients. The radiographic carrying angles were measured on the anterioposterior radiographs according to the method reported by Chapleau et al. [14].

### Cubitus varus

Cubitus varus deformity was determined by the clinical and radiographic carrying angles, by comparing the injured side with the unaffected side at the latest follow-up. Cubitus varus deformity was defined as a difference of > 10° in the clinical or radiographic carrying angle, with the injured side value lower than the unaffected side value.

### Statistical analysis

All variables were analyzed by the SPSS 22.0 statistical software, continuous data were indicated by *X* ± SD, and the Student ANOVA analysis was used for the comparison of continuous variables. Chi-square test was used for categorical variables. The level of statistical significance was determined at *P* < 0.05.

## Results

### Patients demographic data

After screening, there were thirty-two patients included in the present study; among the 32 included patients, there were 27 patients who achieved successful closed reduction under the ultrasound-guided reduction, and 5 patients did not achieve closed reduction and needed further open reduction. The success rate of US-guided closed reduction in the TFDH was 84% (27/32). We enrolled the 27 patients into further analysis. There were 20 males and 7 females. The average age at treatment was 15.39 ± 3.10 months (ranged from 9.40 to 19.43). The average follow-up duration was 34.88 ± 7.15 months (ranged from 24.47 to 49.50). At the last follow-up, the average age was 50.32 ± 7.25 months (ranged from 40.27 to 65.53). The fractures occurred more commonly in the right side (63%, 17 cases) than that in the left side (37%, 10 cases). The demographic information on the included patients is available in Table 1.

**Table 1:** Demographic data of patients

Gender (*n*)	*N* = 27
Male	20 (74.07%)
Female	7 (25.93%)
Laterality (*n*)	
Left	10 (37.04%)
Right	17 (62.96)
The age at injury (months)	15.39 ± 3.10 (9.40–19.43)
Average time to surgery (days)	2.04 ± 1.13 (1–4)
Follow-up (months)	34.88 ± 7.15 (24.47–49.50)
The age at last follow-up (months)	50.32 ± 7.25 (40.27–65.63)

### Clinical outcomes

At the last follow-up, the average flexion of the injured elbow was 145.26 ± 4.94°, and the average extension of the injured elbow was 4.57 ± 4.45°. Differences in elbow flexion were detected between the injured and uninjured side (*P* = 0.027). However, there was no significant difference in the elbow extension between the injured and uninjured side (*P* = 0.110). The mean range of motion was significantly decreased in the injured side when compared to the normal side which was 138.63 ± 5.95° and 143.76 ± 5.95°, respectively (*P* = 0.003). The mean clinical carrying angle at the last follow-up was 11.67 ± 3.11° for the injured side and 10.63 ± 7.16° for the normal side; no difference was detected (*P* = 0.493) (Table 2).

**Table 2:** The functional evaluation of the elbows (affected elbows vs. unaffected elbows)

Outcomes	Degrees (°)	*F*	*P*
Flexion of the affected elbows	145.26 ± 4.94 (136.0–157.0)	5.154	0.027
Flexion of the unaffected elbows	148.33 ± 5.01 (139.0–161.0)		
Extension of the affected elbows	6.63 ± 4.83 (− 1.5–14.0)	2.642	0.110
Extension of the unaffected elbows	4.57 ± 4.45 (− 6.5–10.0)		
ROM of the affected elbows	138.63 ± 5.95 (127.0–150.0)	10.045	0.003
ROM of the unaffected elbows	143.76 ± 5.95 (130.5–155.0)		

The clinical outcomes were classified as excellent, good, fair, or poor according to the Flynn’s criteria. There were two categories of the classification including the loss of motion and the loss of carrying angle in degrees that were compared to the normal elbow. The functional results were excellent in 21 patients (77.78%) and good in 6 (22.22%). The cosmetic results were excellent in 23 patients (85.19 %) and good in 4 (14.81 %). No patients were noted as the fair or poor grade either in the functional and cosmetic evaluation (Table 3).

**Table 3:** Functional and cosmetic outcomes according to Flynn’s criteria (affected elbows vs. unaffected elbows)

Outcomes		Number		*χ*
		Affected elbows	Unaffected elbows	
Functional, loss of range of motion (degrees)	Excellent (0–5)	21 (77.78%)	27 (100.00%)	0.030
	Good (5–10)	6 (22.22%)	0 (0.00%)	
	Fair (10–15)	0 (0.00%)	0 (0.00%)	
	Poor (> 15)	0 (0.00%)	0 (0.00%)	
Cosmetic, difference in carrying angle (degrees)	Excellent (0–5)	23 (85.19%)	27 (100.00%)	0.119
	Good (5–10)	4 (14.81%)	0 (0.00%)	
	Fair (10–15)	0 (0.00%)	0 (0.00%)	
	Poor (> 15)	0 (0.00%)	0 (0.00%)	

### Radiographic outcomes

At the last follow-up, the radiographic carrying angles of both injured elbow and normal elbow were measured. The mean carrying angle was 10.46 ± 3.88° in the injured elbow and 10.48 ± 3.03° in the normal side, respectively. There were no significant difference between the injured side and the unaffected side (*P* = 0.752) (Table 4).

**Table 4:** The radiographic carrying angles (affected elbows vs. unaffected elbows)

	Affected elbows (°)	Unaffected elbows (°)	*F*	*P*
Carrying angle of physical examination	11.37 ± 4.14 (− 4.0–19.0)	10.93 ± 2.93 (2.0–15.0)	0.027	0.651
Carrying angle of radiographs	10.46 ± 3.88 (− 4.20–18.10)	10.48 ± 3.03 (3.20–14.70)	0.020	0.889

### Cubitus varus

Two patients (2/27, 7.4%) showed a cubitus varus deformity according to our criteria aforementioned. The two patients demonstrated the deformity in both radiographs and clinical examination.

## Discussion

Ultrasonography is a noninvasive and rapid available technique which is well applied in the diagnosis of musculoskeletal injuries in infants and young children [15]. Supakul et al. [16] suggested that although the posteromedial displacement of the proximal forearm on the radiography is highly suggestive of TFDH, the definite diagnosis can be confirmed with ultrasound. Dias et al. [17] firstly described the ultrasonic diagnosis of TFDH. They demonstrated the ultrasonography characteristics of the distal humerus that the cartilaginous epiphysis is depicted as a hypoechogenic structure with sparkling echoes within it, whereas the cortical bone appears as a highly echogenic structure with posterior acoustic shadowing. In addition, the ultrasonography is a noninvasive examination without ionizing radiations which could obviate elbow arthrography in the detection of TFDH. More importantly, it can also show the direction and extent of fracture displacement which is essential for the guidance of reduction manipulation [18].

A previous study has reported the US could detect cortical discontinuities of 1 mm or more [19]. Some recent literature also introduced the utilization of ultrasound-guided reduction in forearm and femoral fractures [20–22]. In particular, ultrasonography has the special ability to display the image of the cartilages at the distal part of the humerus in children. Furthermore, intraoperative US-guided reduction provides the image of the fracture displacement continuously in real-time, which help surgeons manipulating the distal segment to reduce without radiation exposure. In the present study, all the fracture reductions were performed under US guidance initially. We achieved a satisfactory successful rate of closed reduction which was 84% (27/32). Some researchers doubted that US may have limited use because of the requirement of significant expertise in performing and interpreting the examination [23, 24]. In fact, we simplified the complicated procedures of US adopted in the diagnostic examination which need multi-plane scanning [15], as the preoperative imaging examination has shown the displacements of the fracture comprehensively. As a result, the aim of intraoperative US is to offer the images to assure the reduction of fracture displacements in the lateral and posteroanterior planes. In the present study, the US in the lateral and posterior plane could adequately and effectively show the structures at fracture site and guide intraoperative reduction.

The cubitus varus deformity seems to be the most common complication after TFDH. Nevertheless, the definite reason of the cubitus varus has not been elucidated. Previous studies reported variable incidences of cubitus varus after treatment of TFDH which ranged from 25 to 70% [25]. In the present series, the incidence of cubitus varus after closed reduction was 7.4%, which is comparable to Hariharan et al.’s recent multicenter review [24]. The lower incidence of the cubitus varus in the present study might be attributed to the following reasons. It has been reported that there is a higher frequency of varus deformity in children less than 2 years of age in the previous studies [26]. However, most patients in the present study were relatively older toddlers with an average age of 15 months which was different from most studies mainly including neonates. Secondly, thanks to the less remodeling potential of the distal humerus, the cubitus varus seems to be not progressive [27]. An inadequate reduction could be the main cause of cubitus varus in toddlers with TFDH [6]. In the present study, all the reduction was guided by the US, and approximately anatomy reduction could be guaranteed in most cases which contributed to the avoidance of the cubitus varus effectively. Thirdly, it has been reported that stable fixation after reduction is also an important factor in preventing cubitus varus [24, 28]. In the present study, considering the maintenance of displaced fracture reduction may be difficult, especially if elbow swelling was presented and then decreased, and plaster immobilization alone could not maintain the reduction stably. All the patients underwent reduction followed by percutaneous pinning fixation. In agreement with previous studies, we deem that this stable fixation procedure was conducive to maintain the position after reduction and further accelerate the callus forming at the fracture site.

There are some limitations in the present study; this is a retrospective study and the follow-up is relatively short; thus, the long-term follow-up of clinical and radiographic outcomes are required to evaluate the development of the cubitus varus. The US could guide the fracture reduction intraoperatively; however, the fixation K-wires were penetrated into the bone which are uneasily delineated by ultrasound. Therefore, radiography is still needed to confirm the configuration of the fixation pins intraoperatively.

In conclusion, our study shows the satisfactory clinical and radiographic outcomes in children with displaced TFDH treated by US-guided closed reduction with percutaneous pining fixation. With the guidance of intraoperative US, the acceptable fracture reduction can be achieved with the advantages of real-time, non-radioactive, and simple utilization.

## Data Availability

The datasets used and/or analyzed during the current study are available from the corresponding author on reasonable request.
